# Congenital perineal hamartomas with rectal duplication: A case report

**DOI:** 10.3389/fmed.2022.1066559

**Published:** 2023-01-12

**Authors:** Yixin Zhang, Mo Zhang, Wei Ma, Zhengwei Yuan, Dajia Wang, Lizhu Chen

**Affiliations:** ^1^Department of Ultrasound, Shengjing Hospital of China Medical University, Shenyang, China; ^2^Department of Urology, The First Hospital of China Medical University, Shenyang, China; ^3^Key Laboratory of Health Ministry for Congenital Malformation, Department of Pediatric Surgery, Shengjing Hospital of China Medical University, Shenyang, China; ^4^Department of Pediatric Surgery, Shengjing Hospital of China Medical University, Shenyang, China

**Keywords:** congenital mass, perineal mass, hamartoma, ultrasound, prenatal

## Abstract

**Background:**

Congenital perineal hamartomas are rare, and reports of prenatal ultrasound diagnosis are limited. Perineal hamartomas are usually associated with other structural malformations, which complicate the therapeutic regime.

**Case presentation:**

We report a case of perineal hamartomas associated with rectal duplication in a female fetus. A review of the literature on similar cases was also presented. A fetus was first diagnosed with a perineal mass at 33 weeks of gestation using ultrasound examination in our hospital. Two-dimensional ultrasonography showed a hyperechoic mass resembling a scrotum in the perineum of the fetus. The pedicle connected the mass to the fetal anus. The masses were excised after birth, and perineal hamartomas were confirmed by pathological diagnosis. Rectal duplication, an associated malformation, was found during the surgery. The rectal duplication cyst was removed at the same time.

**Conclusion:**

Congenital perineal masses are rare and are usually associated with urogenital and anorectal malformations. Prenatal ultrasound should be used to assess the position and relationship between the mass and perineal organs, and to exclude other combined deformities.

## Introduction

According to available reports, congenital perineal masses, including lipomas, hamartomas, and hemangiomas, are rare (1). Although most of them are benign, their unique location and rapid growth characteristics make complete surgical resection the best choice of treatment. Many perineal masses present as ambiguous genitalia that interfere with distinguishing the sex of the fetuses (2). In addition, perineal masses are associated with anorectal and urogenital malformations, which complicate the therapeutic regime (3). Herein, we report a case in which a female fetus was diagnosed with congenital perineal masses that resembled male genital organs on prenatal ultrasound examination. These masses were verified as hamartomas by pathological examination after birth. Rectal duplication, an associated malformation, was found and removed at the same time. Rectal duplication cysts are rare (only 4%) among the congenital gastrointestinal cysts (4), which are described as congenital spherical or tubular cysts located in the presacral space (5).

## Case presentation

**Figure 1:**
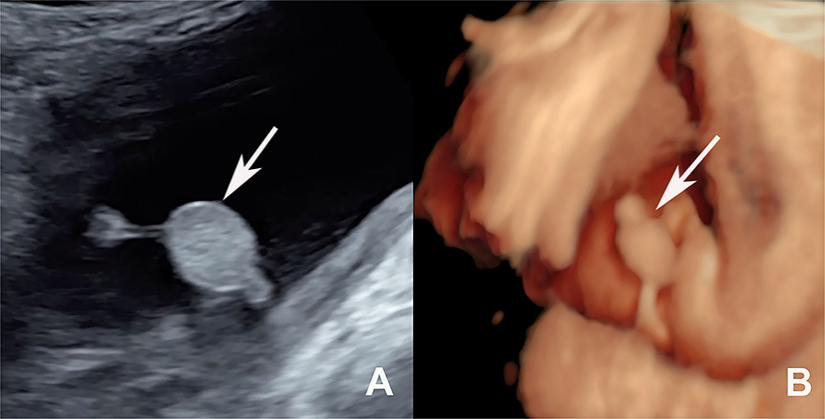
**(A)** Two-dimensional ultrasound showed a hyperechoic mass resembling a scrotum (the white arrow shows) in the perineum of the fetus. **(B)** Three-dimensional ultrasound showed the scrotum-like masses (the white arrow shows) and their relationship with other organs vividly at 33 weeks gestation.

A 34-year-old woman, gravida 2, para 1, was transferred to Shengjing hospital of China Medical University at 33 weeks of gestation. At 24 weeks of pregnancy, the patient was informed that she was pregnant with a boy during an ultrasound examination at the district hospital. However, during the 32-week ultrasound examination, the doctor found abnormalities in the perineum of the fetus. A detailed ultrasound examination was performed at 33 weeks in Shengjing hospital, and a perineal scan revealed that the fetus was female, not male. Two-dimensional ultrasound showed a hyperechoic mass (1.7 × 1.2 cm) resembling a scrotum in the perineum of the fetus (Figure 1A). The pedicle connected the mass to fetal skin around anus. Dynamic observation revealed another small hyperechoic mass (0.8 × 0.7 cm) protruding from the fetal anus, connecting to the other end of the pedicle. The masses floated in the amniotic fluid with no obvious blood flow on color Doppler flow imaging (CDFI). Three-dimensional ultrasound showed scrotum-like masses and their relationship with other organs (Figure 1B). The mother underwent amniocentesis, and no abnormalities were found on a comparative genomic hybridization (CGH) array.

**Figure 2:**
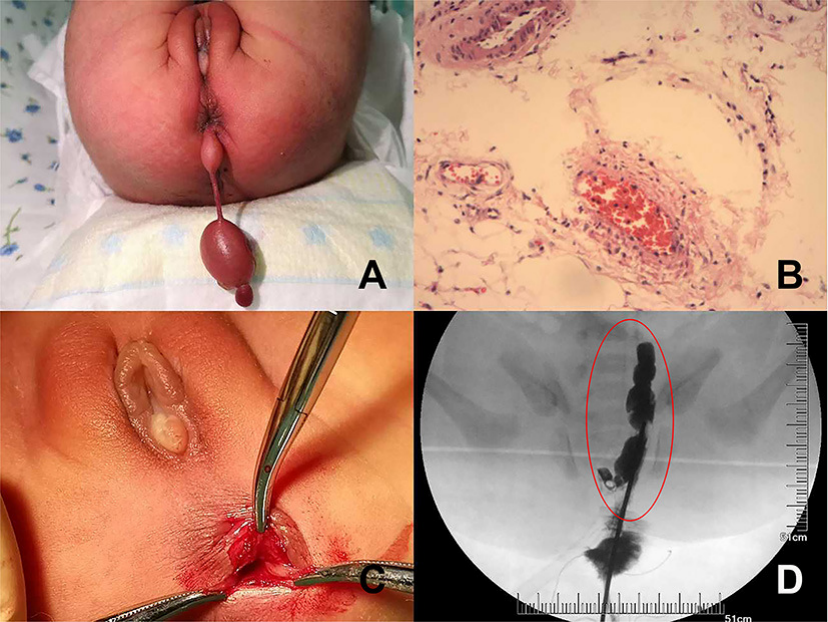
**(A)** Gross appearance of the perineal masses at birth. **(B)** The histological examination results of the perineal masses show hyperplastic lymph vessels and adipose tissue that conformed to hamartomas. **(C)** After the mass was removed, a cavity can be seen clinging to the posterior wall of the rectum. **(D)** Under contrast agent, the radiography shows an irregular cystic mass located in the presacral space.

A female neonate was delivered, weighing 3,240 g at GA 40 + 2 weeks. Her Apgar scores were 10 and 10 at 1 and 5 min, respectively. Perineal examination revealed two soft spherical masses protruding from the patient's anus and connected by a pedicle. The masses were 2.5 × 1.5 cm and 1.4 × 0.7 cm in size (Figure 2A). The neonate underwent an abdominal ultrasound examination, and no other abnormalities were found. We also performed a postnatal ultrasound on the perineal masses. Sonography showed two well-defined hyperechoic masses connected by a pedicle, and CDFI detected blood flow signals in the masses. The results of the sacrococcygeal magnetic resonance imaging (MRI) were normal. Surgery was performed under general anesthesia 1 day after birth. The masses were originated from the peri-anal skin at 4:00 in lithotomy position, and the anal sphincter was not involved. Histopathological examination of the masses suggested hamartomas composed of hyperplastic lymph vessels and adipose tissues (Figure 2B). After the masses were removed, a cavity was discovered along the posterior rectal wall (Figure 2C). The contrast agent showed an irregular cystic mass connected to the posterior wall of the rectum, and rectal duplication was suspected (Figure 2D). An *en bloc* excision of the cystic mass and the posterior wall of the rectum was performed. Rectal duplication was confirmed by histopathological examination. At the time of writing this report, the patient was 2.5 years old, and recovered well after surgery without other abnormalities.

## Discussion

Perineal masses include lipomas, hamartomas, hemangiomas, and other types of tumors (1). Perineal hamartomas are rarely found in the fetus; however, their prenatal ultrasound appearance is not significantly different from other benign perineal masses like lipomas. Perineal masses often interfere with the identification of the sex of the fetus and are sometimes misdiagnosed as ambiguous genitalia (AG) (2). AG is a morphological diagnosis defined as genitalia not typical of a male or female. Findings mimicking AG, such as penoscrotal anomalies, anorectal malformations, and perineal tumors, may prevent accurate identification of the fetal sex (6). In our case, the perineal hamartomas mimicked the male scrotum, thus confusing sex diagnosis. As a result of the misdiagnosis, the perineal mass was missed in the second-trimester ultrasound examination. After careful scanning, we found that the fetus had normal female external genitalia, and the so-called “scrotum” originated from the fetus' anus. Thus, prenatal examination of the genitalia should be performed carefully to avoid missed diagnosis of fetal sex and perineal mass.

Only a few studies have reported congenital perineal masses. We found 23 similar cases (including our case) are summarized in Table 1. Histopathological diagnoses included lipomas (17/23) and hamartomas (6/23). The chromosomal examinations were normal in 8 cases and 14 cases were not described. In our case, the chromosomal examination was normal. Fourteen cases (60.9%) had urogenital or anorectal defects, including imperforate anus (1, 6, 10), rectovestibular fistula, rectovesical fistula, rectourethral fistula, urogenital sinus, penoscrotal hypospadias, penoscrotal transposition, vesicoureteral reflux (3), vaginal duplex, uterus duplex (8), bifid scrotum (6, 11), accessory scrotum (15), and so on. Therefore, the appearance of a perineal mass might serve as a marker for predicting anorectal defects or urogenital defects. Most perineal masses are benign tumors that can be radically excised; however, surgery will be complicated if there are other associated malformations. Therefore, the prenatal detection of other associated malformations is critical for prognostic assessment. In our case, we performed a comprehensive scanning of the perineal organs. There were no other abnormalities *in utero* on comprehensive sonographic scanning and no other abnormalities on MRI after birth. However, rectal duplication was observed during postnatal surgery. This suggests that prenatal ultrasound examination was limited to the examination of the perineal organs and structures. Pediatric surgeons should be aware of the possibility of other associated malformations, even if prenatal examinations are normal.

**Table 1:** A summary of the literature review findings for congenital perineal masses.

**Report**	**Sex**		**Mass quantity and size (n; cm)**	**Histopathology**	**Chromosomal examination**	**Associated malformations**
	**Male**	**Female**				
1 (6)	1	0	1; 2.8 × 1.5 cm	Lipoma	Normal	Bifid scrotum, imperforate anus with a fistula
2 (7)	1	0	1; 3.4 × 2.0 cm	Hamartoma	Not described	No other malformations
3 (8)	0	1	1; 5.0 × 3.0 cm	Hamartoma	Normal	Vaginal duplex, uterus duplex
4 (9)	1	0	1; 2.0 cm	Lipoma	Not described	No other malformations
5 (10)	0	2	Case 1, 1; 3.0 × 2.0 cm Case 2, 1; Not described	Both hamartomas	Case 1, Not described; Case 2, Not described	Imperforate anus (Case 1, female); No other malformations (Case 2, female)
6 (3)	3	3	Cases 1–6, 6; Cases 1–6, Not described	All lipomas	Cases 1–6, Not described	A high cloaca, a short and small vagina (Case 1, female); A rectourethral fistula and penoscrotal hypospadias (Case 2, male); A rectovestibular fistula (Case 3, female); A high anorectal malformation, penoscrotal transposition, and midshaft hypospadias (Case 4, male); A rectovestibular fistula, urogenital sinus and bilateral Grade V vesicoureteral reflux (Case 5, female); A high anorectal malformation, rectovesical fistula, and hemisacral defect (Case 6, male)
7 (11)	1	0	1; 6.0 × 5.0 × 3.0 cm	Hamartoma	Normal	Bifid scrotum, anorectal malformation
8 (12)	0	1	1; 3.0 × 2.1 × 2.0 cm	Lipoblastoma	Normal	No other malformations
9 (13)	1	0	1; 5.0 × 1.0 cm	Lipoma	Not described	No other malformations
10 (14)	0	1	1; 4.0 cm	Lipoma	Not described	No other malformations
11 (15)	1	1	Case 1, 1; 3.5 × 2.0 cm Case 2, 2; 2.5 × 2.0 cm and 1.0 × 0.9 cm	Both lipomas	Case 1: Not described Case 2: Not described	No other malformations (Case 1, female); Accessory scrotum (Case 2, male)
12 (16)	0	1	1; 5.0 × 4.0 × 3.0 cm	Lipoma	Normal	No other malformations
13 (1)	0	2	Case 1, 1; 3.0 × 2.0 × 1.5 cm Case 2, 1; 1.5 × 1.5 × 1.0 cm	Both lipomas	Case 1: Normal Case 2: Normal	No other malformations (Case 1, female); Imperforate anus (Case 2, female)
14 (17)	0	1	1; Not described	Lipoma	Normal	Accessory phallic urethra and anterior ectopic anus
Our case	0	1	2; 1.4 × 0.7 cm and 2.0 × 1.5 cm	Hamartoma	Normal	Rectal duplication
Total	9	14	/	Lipoma: 17 Hamartoma: 6	Not described: 14 Normal: 9	No malformations: 9 Anorectal malformations and/or urogenital malformations: 14

Here, we report a case of perineal hamartomas associated with rectal duplication, for the first time. Rectal duplication is uncommon. Patients with rectal duplication present with chronic constipation, acute urine retention, and other clinical manifestations (18). In our case, the perineal masses and rectal duplication were removed simultaneously 1 day after birth, and the neonate was followed up to 2.5 years of age without obvious symptoms.

The embryogenesis of congenital perineal masses can be poorly interpreted. However, congenital perineal masses can destroy the continuity of the sacrococcygeal region, leading to combined defects of the anorectal and genitourinary organ systems (15, 19). In addition, since the embryologic development of the urogenital sinus, rectum, anus, and perineum are closely related, the associated anomalies in cases with perineal masses can be explained (8).

Three-dimensional ultrasound plays an important role in auxiliary diagnosis. We used the three-dimensional surface imaging technology to vividly display the perineal masses, which also clearly showed the origin and location of the mass and its relationship with other organs. Three-dimensional ultrasound is also conducive to enhancing diagnostic confidence and facilitating better communication with patients.

## Conclusion

In conclusion, prenatal ultrasound examination plays a leading role in distinguishing the origin of perineal hamartomas and evaluating the associated abnormalities. This could provide an efficient basis for clinical counseling and postnatal treatment.

## Data availability statement

The original contributions presented in the study are included in the article/supplementary material, further inquiries can be directed to the corresponding authors.

## Ethics statement

Written informed consent was obtained from the individual(s), and minor(s)' legal guardian/next of kin, for the publication of any potentially identifiable images or data included in this article.

## Author contributions

YZ and LC were responsible for the original draft preparation and editing. MZ, WM, and YZ participated in data collection. LC, DW, and ZY were responsible for conceptualization, manuscript editing, and manuscript review. All authors have read and approved the final manuscript.
